# Factors Related to the Growth of Oral Bacteria After Surgery: An Observational Study of 54 Patients Undergoing Oncologic or Cardiac Surgery

**DOI:** 10.7759/cureus.72342

**Published:** 2024-10-24

**Authors:** Madoka Funahara, Sakiko Soutome, Yuki Sakamoto, Akira Imakiire, Mitsunobu Otsuru, Masahiro Umeda

**Affiliations:** 1 School of Oral Health Sciences, Faculty of Dentistry, Kyushu Dental University, Kitakyusyu, JPN; 2 Department of Oral Health, Nagasaki University Graduate School of Biomedical Sciences, Nagasaki, JPN; 3 Department of Oral Surgery, Kansai Medical University Medical Center, Moriguchi, JPN; 4 Department of Oral and Maxillofacial Surgery, Kanagawa Dental University, Yokosuka, JPN; 5 Department of Research and Treatment for Oral and Maxillofacial Congenital Anomalies, School of Dentistry, Aichi Gakuin University, Nagoya, JPN; 6 Department of Clinical Oral Oncology, Nagasaki University, Nagasaki, JPN

**Keywords:** dental plaque, number of bacteria in saliva, oral feeding, perioperative oral management, postoperative pneumonia

## Abstract

Introduction

Postoperative complications, such as surgical site infection and postoperative pneumonia, may be caused by oral bacteria. This study aimed to clarify the factors related to the bacterial count in the saliva of postoperative patients so as to standardize oral management methods before and after surgery.

Methods

This prospective observational study enrolled 54 patients who underwent major oncologic or cardiac surgery. The following variables were investigated: age, sex, primary disease, body mass index, performance status, smoking, alcohol consumption, serum creatinine and albumin, operation time, intraoperative blood loss, number of teeth, functional tooth unit, plaque control record (PCR), amount of dental plaque, community periodontal index, eating status the day after surgery, oral wetness, and number of bacteria in the saliva before and after surgery. The relationship between each variable and the number of bacteria in the saliva before and after surgery was analyzed.

Results

Multiple regression analysis revealed that the PCR was significantly associated with the number of bacteria in the saliva before surgery (p=0.021). On the day after surgery, the number of bacteria in saliva was significantly higher than that before surgery (p<0.001). Multivariate analysis showed that eating status the day after surgery (p=0.046) and oral wetness (p=0.043) were significantly associated with the number of bacteria, but dental plaque did not influence the bacterial count.

Conclusions

Postoperative salivary bacterial counts increased due to reduced oral self-cleaning, regardless of dental plaque content. Therefore, oral feeding should be started promptly after surgery to reduce the number of bacteria in the saliva.

## Introduction

Complications such as surgical site infection (SSI) and postoperative pneumonia may occur after highly invasive surgery. Some are thought to be caused by oral bacteria, and dental treatment and oral care are recommended before and after surgery to reduce the risk of these complications. There are two possible mechanisms by which oral infections cause infectious complications: blood circulation infections of oral bacteria and direct exposure to saliva, including oral bacteria. SSIs in head and neck cancer surgery and upper gastrointestinal cancer surgery are caused by direct contact between saliva-containing oral bacteria and the wound. In addition, postoperative pneumonia may occur due to aspiration of saliva containing pathogenic microorganisms in postoperative dysphagia-prone surgery, such as esophageal cancer surgery. Therefore, it is necessary to reduce the number of bacteria in the saliva after surgery to prevent complications.

There are many reports that the main source of bacteria in saliva is bacteria in dental plaque or periodontal pockets. Mojon reported that the mechanisms of pneumonia could be the colonization of dental plaque by respiratory pathogens, followed by aspiration or colonization of periodontal pathogens [1]. Sumi et al. stated that bacteria that commonly cause respiratory infection colonize the dental plaque of older adult dependent subjects; therefore, dental plaque must be considered a specific reservoir of colonization and subsequent aspiration pneumonia in the dependent older adults [2]. Scannapieco et al. reported that bacteria that cause nosocomial pneumonia colonize the dental plaque of intensive care unit (ICU) patients, and dental plaque may be an important reservoir of these pathogens [3]. Fourrier et al. also concluded that dental plaque must be considered a specific reservoir of colonization and subsequent nosocomial infection in ICU patients [4].

However, randomized controlled trials indicated that tooth brushing did not reduce the risk of ventilator-associated pneumonia in patients under intubation [5-7]. Furthermore, the US Institute for Healthcare Improvement recommends daily oral disinfection using 0.12% chlorhexidine to prevent pneumonia in patients with mechanical intubation; however, tooth brushing is not described [8]. We previously reported that the number of bacteria in the saliva increased significantly on the day after surgery, although the amount of dental plaque was not increased compared to that before the operation; therefore, the number of bacteria in the saliva was not related to dental plaque [9].

In Japan, perioperative oral management has been covered by public health insurance since 2014, and the preoperative removal of dental plaque and calculus is widespread in many hospitals; however, dental interventions immediately after surgery are less common. The purpose of this study was to clarify the factors related to the number of bacteria in the saliva of postoperative patients in order to standardize oral management methods before and after surgery.

## Materials and methods

Patients

This study was a prospective observational study designed to clarify the factors related to the number of bacteria in the saliva of postoperative patients. The inclusion criteria were patients who underwent major oncology or cardiothoracic surgery at Nagasaki University Hospital, Nagasaki, Japan, between January and March 2017 and were referred for preoperative oral care. The exclusion criteria are patients under 20 years of age or patients with severe xerostomia who have difficulty collecting saliva samples.

Intervention

Patients who participated in the study received standard preoperative oral care from dentists and dental hygienists. Oral care was initiated at the time of admission. The contents of the dental intervention included oral hygiene guidance, removal of dental calculus (scaling) and dental plaque, removal of tongue coating with a toothbrush, cleaning of dentures, extraction of infectious teeth with apical lesion ≥ 3mm or periodontal pocket ≥ 8mm, and clinical symptoms such as swelling, pain, and pus discharge, and instructions for gargling.

Variables

The following variables were investigated before surgery: demographic factors like age, sex, smoking, and alcohol habit; oral health: number of teeth present, functional tooth unit (FTU), plaque control record (PCR), amount of dental plaque, community periodontal index, oral wetness, and number of bacteria in the saliva; laboratory values: preoperative serum creatinine, preoperative serum albumin. Other variables related to surgery: primary disease, Eastern Cooperative Oncology Group (ECOG) performance status (PS), and body mass index (BMI). The number of bacteria in the saliva was also examined the next day after surgery.

Smoking and alcohol habits were considered present if the patient had done either within the past year. PS was divided according to the Eastern Cooperative Oncology Group criteria. Standardized criteria for measuring how much a disease affects a patient's ability to perform daily activities is classified into five levels from Grade 0 (normal) to Grade 4 (totally immobile) [10]. The number of FTUs was defined according to the number of opposing premolars or molars as follows: two opposing premolars were defined as one FTU and two opposing molars were defined as two FTUs [11]. Therefore, if all premolars and molars are aligned, the number of FTUs was 12. FTUs are classified into two types: n-FTU and nif-FTU. Specifically, n-FTUs refer to FTUs of natural teeth, and nif-FTUs refer to FTUs of natural teeth and artificial teeth on implant-supported or fixed prostheses. The PCR was performed using the O’Leary plaque record [12]. The amount of dental plaque was an index we defined that reflects the amount of dental plaque in the oral cavity and is calculated by multiplying the O'Leary plaque score by the number of teeth present [13]. The community periodontal index was defined as follows [14]: code 0 for teeth with normal periodontal pockets, code 1 for teeth with pockets of 4-5 mm, code 2 for teeth with pockets <6 mm, code 9 for teeth that cannot be probed, and code X for no corresponding teeth. Oral wetness was measured at the surface of the buccal mucosa using an oral hydrometer (Moisture Checker Mucus®, Life Co., Ltd., Saitama, Japan). The number of bacteria in the saliva was measured according to a previously reported method with a rapid oral bacteria quantification system (Panasonic Healthcare Co. Ltd., Osaka, Japan) using dielectrophoresis and impedance measurement methods [15,16]. The number of bacteria in saliva may be influenced by eating. Therefore, before surgery, saliva samples were collected before and after oral care, avoiding one hour after meal intake. After surgery, saliva was collected before breakfast and before oral care.

Statistical analysis

All statistical analyses were performed using IBM SPSS Statistics for Windows, Version 26 (Released 2019; IBM Corp., Armonk, New York, United States). The relationship between each variable and the number of bacteria in the saliva after surgery was analyzed. A one-way analysis of variance was used for the categorical variables, and Spearman's rank correlation coefficient was used for continuous variables. The multivariate analysis, all factors were entered, the model that fits best was created using a stepwise method, and multiple regression analysis was performed.

## Results

Patient characteristics

Fifty-four patients who underwent oncologic or cardiac surgery and received oral care prior to surgery were enrolled. The characteristics of the participants are summarized in Table 1 and Table 2. There were 32 men and 22 women, with an average age of 67.4 years. Forty-four patients underwent major oncologic surgery, and 10 underwent cardiac surgery. The eating status the day after surgery was oral feeding in 10, fasting in 40, and no feeding due to intubation in four patients.

**Table 1:** Patient characteristics of the categorical variable Categorical variable on 54 people who received oral care before surgery. PS: Performance Status by Eastern Cooperative Oncology Group criteria; CPI: community periodontal index

Variable			Number of patients
Sex	Male		32
	Female		22
PS	PS 0		43
	PS 1		7
	PS 2		4
Smoking habit	(-)		48
	(+)		6
Drinking habit	(-)		41
	(+)		13
Periodontal disease	CPI=0 or 9		31
	CPI=1 or 2		23
Surgery	Oncologic surgery		44
	Gastrointestinal cancer		17
	Lung cancer		14
	Breast cancer		5
	Head and neck cancer		4
	Kidney cancer		2
	Prostate cancer		1
	Retroperitoneal cancer		1
	Cardiac surgery		10
Eating status the day after surgery	Oral feeding		10
	Fasting		40
	Intubation		4

**Table 2:** Patient characteristics of continuous variable Continuous variable on 54 people who received oral care before surgery. n-FTU: functional teeth unit of natural teeth; nif-FTU: functional teeth unit of natural teeth and artificial teeth on implant-supported or fixed pontics

Variable	mean±SD
Age (years)	67.4±14.5
Body mass index (kg/m^2^)	22.0±3.33
Albumin (g/dL)	4.04±0.617
Creatinine (mg/dL)	0.833±0.262
Oral wetness	26.1±3.33
Plaque control record (%)	29.6±25.1
Number of teeth	18.2±10.3
Amount of dental plaque	499±384
n-FTU	5.15±4.83
nif-FTU	5.70±5.16
Operation time (minutes)	278±177
Blood loss (g×10^2^)	3.84±7.64

Changes in the number of bacteria in saliva before and after surgery

**Figure 1:**
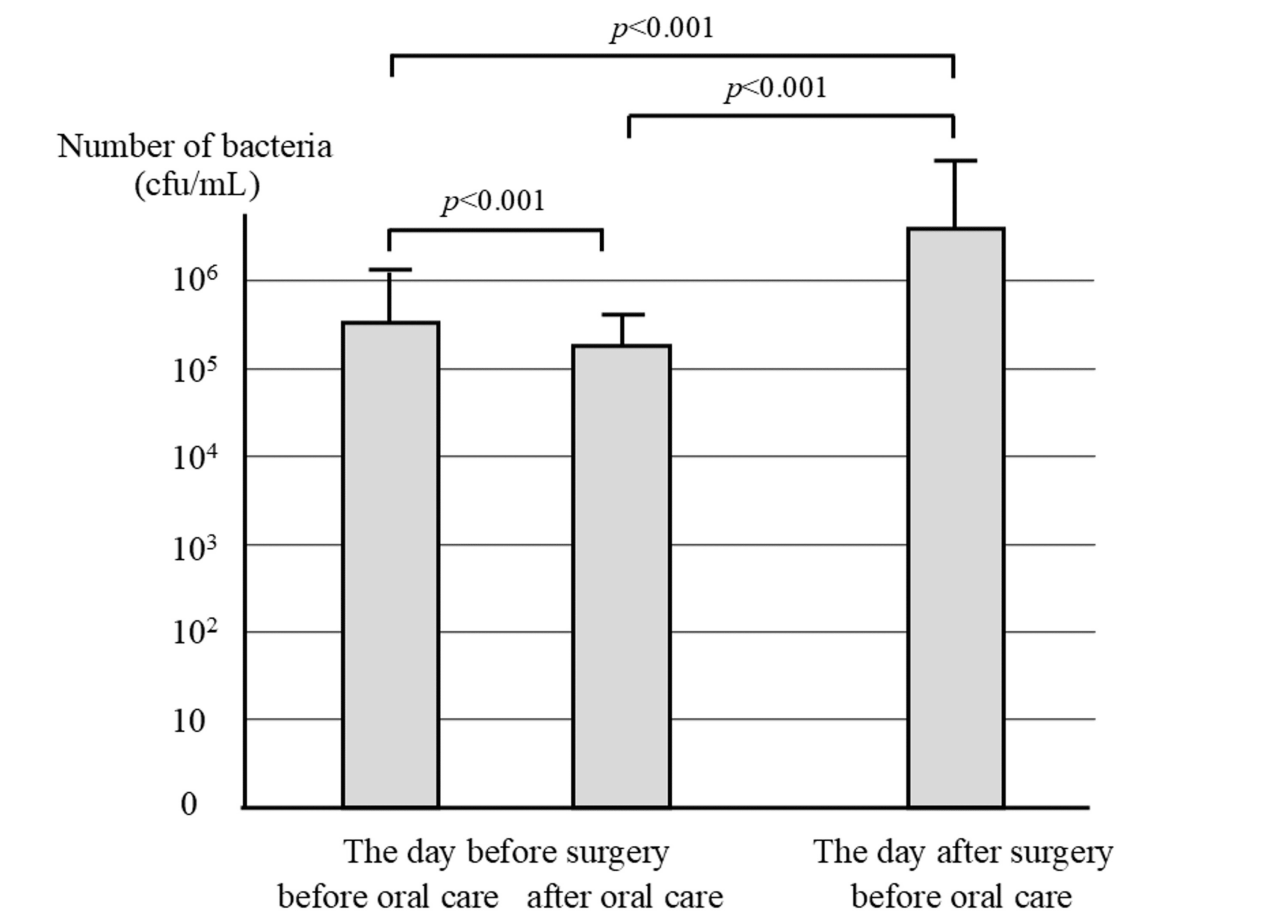
Changes in the number of bacteria in saliva Number of bacteria in saliva before and after oral care on the day before and the day after surgery.

The logarithm of the number of bacteria in saliva before oral care before surgery was 5.54 ± 0.52. Oral care significantly decreased it to 5.27 ± 0.39. However, after surgery, it significantly increased to 6.61 ± 0.91 (Figure 1).

Factors related to the number of bacteria in saliva the day before surgery and before oral care

Univariate analysis revealed that the PCR (p=0.003) and amount of dental plaque (p=0.018) were significantly correlated with the number of salivary bacteria before oral care the day before surgery (Table 3 and Table 4). Multivariate analysis was performed by adding the PCR, number of teeth, and oral wetness as covariates. Only the PCR was significantly associated with the number of bacteria in the saliva (p=0.021) (Table 5).

**Table 3:** Factors related to the number of bacteria in saliva the day before surgery (univariate analysis of categorical variable) The univariate analysis of the categorical variable showed no significant factors related to the number of bacteria in the saliva before preoperative oral care. § One way ANOVA (*p<0.05, **p<0.01.) PS: Performance Status by Eastern Cooperative Oncology Group criteria; CPI: community periodontal index

Variable		Number of total bacteria (logarithm)	p-value §
Sex	Male	5.45±0.471	0.097
	Female	5.68±0.120	
PS	PS 0	5.50±0.469	0.275
	PS 1-2	5.70±0.689	
Smoking habit	(-)	5.51±0.527	0.230
	(+)	5.79±0.412	
Drinking habit	(-)	5.51±0.524	0.433
	(+)	5.64±0.515	
Primary disease	Heart disease	5.39±0.423	0.298
	Cancer	5.58±0.520	
Periodontal disease	CPI=0 or 9	5.55±0.540	0.958
	CPI=1 or 2	5.54±0.502	

**Table 4:** Factors related to the number of bacteria in the saliva the day before surgery (univariate analysis of continuous variable) The univariate analysis of the continuous variable, the relationship between the number of bacteria in saliva before surgery, and the variable revealed significant differences in plaque control record and the amount of dental plaque. † Spearman's rank correlation coefficient (*p<0.05, **p<0.01.) n-FTU: functional teeth unit of natural teeth; nif-FTU: functional teeth unit of natural teeth and artificial teeth on implant-supported or fixed pontics

Variable		Spearman's correlation coefficient	p-value †
Age		0.056	0.686
Body mass index		-0.105	0.452
Albumin		0.080	0.570
Creatinine		0.025	0.857
Oral wetness		0.240	0.081
Plaque control record		0.397	**0.003
Amount of dental plaque		0.321	**0.018
Number of teeth		0.028	0.842
n-FTU		-0.036	0.795
nif-FTU		-0.001	0.938

**Table 5:** Factors related to the number of bacteria in the saliva the day before surgery (multivariate analysis) Multiple regression analysis (*p<0.05), stepwise selection. Multivariate analysis of the number of bacteria in saliva before surgery, plaque control record, number of teeth, and oral wetness identified plaque control record as a significant factor.

Variable	Unstandardized coef.		Standardized coef.	95% confidence interval		p-value
	B	SE	β	lower	upper	
Oral wetness	0.029	0.024	0.162	-0.020	0.077	0.243
Number of teeth	0.005	0.007	0.101	-0.009	0.019	0.474
Plaque control record	0.007	0.003	0.322	0.001	0.012	*0.021

Factors related to the number of bacteria in the saliva the day after surgery

The results of the univariate analysis showed that the eating status the day after surgery (p=0.001), oral wetness (p=0.031), number of teeth (p<0.001), n-FTU (p<0.001), nif-FTU (p<0.001), operation time (p=0.003), and blood loss (p=0.001) were significantly related to the number of bacteria in the saliva the day after surgery (Table 6 and Table 7). Subsequently, in the multivariate analysis with the PCR, eating status the day after surgery, number of teeth, oral wetness, and operation time were as covariates; oral wetness (p=0.043) and the eating status the day after surgery (p=0.046) were significantly associated with the number of bacteria in the saliva (Table 8).

**Table 6:** Factors related to the number of bacteria in the saliva the day after surgery (univariate analysis of categorical variable) Univariate analysis of the categorical variable, the number of bacteria in saliva on the day after surgery, and the variables revealed significant differences in the eating status the day after surgery. § One-way ANOVA (*p<0.05, **p<0.01.) PS: Performance Status by Eastern Cooperative Oncology Group criteria; CPI: community periodontal index

Variable (categorical variable)		Number of total bacteria (logarithm)	p-value §
Sex	Male	6.67±0.929	0.791
	Female	6.59±0.912	
PS	PS 0	6.60±0.923	0.841
	PS 1-2	6.67±0.887	
Smoking habit	(-)	6.65±0.889	0.433
	(+)	6.34±1.09	
Drinking habit	(-)	6.59±0.912	0.791
	(+)	6.67±0.929	
Primary disease	Heart disease	7.00±1.01	0.193
	Cancer	6.54±0.881	
Periodontal disease	CPI=0 or 9	6.66±0.877	0.698
	CPI=1 or 2	6.55±0.967	
Eating status the day after surgery	Oral feeding	5.84±0.801	**<0.001
	Fasting	6.67±0.805	
	Intubation	7.86±0.908	

**Table 7:** Factors related to the number of bacteria in the saliva the day after surgery (univariate analysis of continuous variable) Univariate analysis of the number of bacteria in saliva on the day after surgery and the variables revealed significant differences in the oral wetness, number of teeth, n-FTU, nif-FTU, operation time, and blood loss. † Spearman's rank correlation coefficient (*p<0.05, **p<0.01.) n-FTU: functional teeth unit of natural teeth; nif-FTU: functional teeth unit of natural teeth and artificial teeth on implant-supported or fixed pontics

Variable	Spearman's correlation coefficient	p-value †
Age	0.266	0.062
Body mass index	-0.055	0.702
Albumin	-0.054	0.709
Creatinine	0.201	0.161
Oral wetness	-0.306	*0.031
Plaque control record	0.145	0.316
Amount of dental plaque	-0.089	0.537
Number of teeth	-0.590	**<0.001
n-FTU	-0.511	**<0.001
nif-FTU	-0.492	**<0.001
Operation time	0.415	**0.003
Blood loss	0.470	**0.001
Number of bacteria before surgery	0.017	0.907

**Table 8:** Factors related to the number of bacteria in the saliva the day after surgery (multivariate analysis). Multiple regression analysis (*p<0.05), stepwise selection. Multivariate analysis of the number of bacteria in saliva on the day after surgery, plaque control record, eating status the day after surgery, number of teeth, oral wetness, and operation time identified oral wetness and the eating status the day after surgery as significant factors.

Variable	Unstandardized coef.		Standardized coef.	95% confidence interval		p-value
	B	SE	β	lower	upper	
Oral wetness	-0.076	0.036	-0.243	-0.149	-0.001	*0.043
Plaque control record	0.002	0.004	0.061	-0.006	0.010	0.601
Number of teeth	-0.022	0.011	-0.255	-0.045	0.001	0.056
Operation time	0.11	0.066	0.214	-0.023	0.243	0.102
Eating status the day after surgery	0.498	0.243	0.277	0.009	0.987	*0.046
Multiple regression analysis (*p<0.05)						

## Discussion

This study aimed to clarify the factors related to the number of bacteria in the saliva of postoperative patients in order to standardize oral management methods before and after surgery. The results indicated that the number of bacteria in the saliva increased significantly after surgery. Furthermore, the increase in the salivary bacterial count was related to a poor PCR before surgery, but after surgery, the PCR and amount of dental plaque were not related to the bacterial count, and inability to eat orally, dry mouth, and loss of teeth were significantly associated with an increase in the number of bacteria in the saliva.

Many studies have reported that plaque and bacteria in the periodontal pocket are sources of bacteria in the saliva and that aspiration of these bacteria is one of the causes of pneumonia in intubated patients or older adults [1-4]. These studies demonstrated that plaque- and periodontal pocket-derived bacteria were present in the sputum and therefore caused aspiration pneumonia. Based on these results, oral care by dentists and dental hygienists, including removal of dental plaque and calculus and professional mechanical tooth cleaning, has been widely performed before highly invasive surgery or for older adults with weakened immunity.

Yoneyama et al. reported that weekly professional oral care by dentists and dental hygienists reduced the incidence of pneumonia in older adult individuals requiring long-term care in a randomized controlled trial [17]. However, in their study, not only weekly professional oral care but also post-meal toothbrushing by caregivers and gargling, and in some cases oral cleaning with povidone-iodine, were performed simultaneously in the intervention group but not in the control group. Furthermore, the incidence of pneumonia in older adults with teeth was 21% in the control group and 9% in the intervention group, while that in edentulous older adults who had no dental plaque or periodontal pockets because they had no teeth was 20% in the control group. Thus, there was no difference in the incidence of pneumonia between those with and without teeth, which suggested that the cause of pneumonia was not plaque or periodontal pockets. These findings suggest that it is not weekly professional oral care but toothbrushing and gargling after each meal and wiping with povidone-iodine that are effective in preventing aspiration pneumonia in older adults.

We previously found that in patients undergoing postoperative ventilator management, the number of bacteria in the saliva after surgery was over ten-fold that before surgery, even though there was no difference in dental plaque adhesion before and after surgery [9]. In patients undergoing intubation management from tracheotomy after oral cancer surgery, the number of bacteria in the oropharyngeal fluid was also increased over 100-fold compared to that before surgery [18]. These results suggest that the increase in the bacterial count was not caused by dental plaque but by a decrease in the self-cleaning effect of the oral cavity due to intubation.

Saliva secretion and swallowing function are important for the self-cleaning effect of the oral cavity. Saliva dilutes the concentration of bacteria, and swallowing flushes the bacteria out of the oral cavity. To promote saliva secretion, mastication is required, and the number of functional teeth and tongue pressure, which enable movement of the bolus, play important roles in mastication. Many investigators have stated that tongue pressure decreases as the flail of the body progresses, but in some cases, tongue pressure is often maintained even if the muscle strength of the body decreases. This is because oral feeding, particularly chewing required for a regular diet, trains the muscles of the tongue, and it is considered that if many teeth are lost, chewing ability is reduced, which in turn reduces tongue pressure. We previously reported that tongue pressure decreased as the FTU decreased in older adults [19]. We confirmed using videoendoscopic (VE) examination that in hospitalized older adults, a decrease in tongue pressure was associated with a decline in swallowing function and was also linked to pneumonia-related death [20]. Additionally, we found that a decrease in tongue pressure was associated with an increase in salivary bacterial counts in older adults [21].

From the results of this study, the number of bacteria in the saliva before surgery increased in patients with poor PCRs. It is probable that the oral hygiene condition was reflected in the number of bacteria in the saliva because all the preoperative patients were orally fed a regular diet and the self-cleaning effect of the oral cavity was maintained. However, many patients fasted the day after surgery, and the self-cleaning effect in the oral cavity decreased, resulting in a higher number of bacteria in the saliva, regardless of the amount of dental plaque. It was found that the feeding status and oral wetness were significant factors influencing the bacterial count in the saliva, and the number of teeth also tended to be associated with the bacterial count, albeit not significantly, while dental plaque was not associated with the bacterial count. Based on the above results, oral feeding should be started as soon as possible after surgery to reduce the number of bacteria in the saliva after surgery, and frequent gargling may be effective during the fasting period.

This study has some limitations. First, this was an observational study with a small number of patients; thus, unknown confounding factors may exist. Second, since the endpoint was the number of bacteria in the saliva, it was unclear whether this factor actually affected the development of postoperative complications. However, in perioperative oral care, there have been no studies focusing on oral feeding, the number of teeth, and oral wetness instead of removing dental plaque and calculus. In the future, we would like to investigate whether postoperative gargling can suppress the increase in the number of oral bacteria and reduce the risk of postoperative complications in a larger number of cases.

## Conclusions

In this prospective observational study, 54 patients who underwent major oncologic or cardiac surgery were enrolled to analyze the relationship between each variable and the salivary bacterial count before and after surgery. Postoperatively, a reduction in oral self-cleaning led to an increase in salivary bacterial counts, irrespective of dental plaque presence. Therefore, it is recommended that oral feeding be initiated promptly after surgery to reduce salivary bacterial counts.
